# Functional Identification of the Terpene Synthase Family Involved in Biosynthesis in *Paeonia lactiflora*

**DOI:** 10.3390/molecules29194662

**Published:** 2024-09-30

**Authors:** Yufeng Zhao, Guanghong Cui, Jian Wang, Ying Ma, Yang Han, Ping Su, Juan Guo, Jiyu Zhang, Luqi Huang

**Affiliations:** 1 State Key Laboratory of Grassland Agro-Ecosystems, Key Laboratory of Grassland Livestock Industry Innovation, Ministry of Agriculture and Rural Affairs, College of Pastoral Agriculture Science and Technology, Lanzhou University, Lanzhou 730020, China; zhaoyf20@lzu.edu.cn; 2 State Key Laboratory for Quality Ensurance and Sustainable Use of Dao-di Herbs, National Resource Center for Chinese Materia Medica, China Academy of Chinese Medical Sciences, No. 16 Dongzhimennei Nanxiao Street, Dongcheng District, Beijing 100700, China; guanghongcui@163.com (G.C.); jianwang2021@126.com (J.W.); xiaoma1110@126.com (Y.M.); hanyang0103@126.com (Y.H.); suping@nrc.ac.cn (P.S.); guojuanzy@163.com (J.G.)

**Keywords:** *Paeonia lactiflora*, paeoniflorin, terpene synthases, monoterpene, sesquiterpene

## Abstract

The root of *Paeonia lactiflora* pall. is a significant component of traditional Chinese medicine, with terpenoids and their glycosides, such as paeoniflorins, serving as key active ingredients known for their anti-inflammatory, hepatoprotective, and analgesic properties. By generating a transcriptome and functionally characterizing 32 terpene synthases (TPSs) from *P. lactiflora*, we successfully constructed 24 pESC-Trp-PlTPS expression vectors. Through expression in *Saccharomyces cerevisiae* engineered strains, we identified four mono-TPSs and five sesqui-TPSs that produce 18 compounds, including eight monoterpenes and ten sesquiterpenes in vitro. This includes a bifunctional enzyme (PlTPS22). Additionally, PlTPS21 was characterized as a pinene synthase with α-pinene as its main product. The expression pattern of PlTPS21 aligns closely with the accumulation patterns of paeoniflorins and α-pinene in the plant, suggesting that PlTPS21 is a key enzyme in the biosynthesis of paeoniflorin.

## 1. Introduction

*Paeonia lactiflora* Pall. (commonly known as shaoyao) is a perennial medicinal shrub of the genus Paeonia. It is categorized into: PAEONIAE RADIX RUBRA (also known as chishao or red peony root) and PAEONIAE RADIX ALBA (also known as baishao or white peony root) [1]. This plant has been used in traditional Chinese medicine for over 2000 years in Asia [2,3,4], with broad pharmacological activities (e.g., anti-inflammatory, analgesic, and antispasmodic effects) to treat conditions like rheumatism, menstrual disorders, and muscle pain [5,6,7]. The medicinal value of *P. lactiflora* is attributed to its rich chemical composition, which includes over 180 identified compounds, such as monoterpenoids, sesquiterpenes, flavonoids, tannins, stilbenes, triterpenoids, steroids, and phenols [8]. Among these compounds, monoterpenoids are particularly significant due to their pharmacological efficacy. Paeoniflorins, a type of pinene monoterpenoid found exclusively in some *Paeonia* species, are the primary active ingredients. These compounds, including paeoniflorin, albiflorin, and benzoyl paeoniflorin, exhibit diverse pharmacological activities, such as anti-inflammatory, antioxidant, and neuroprotective effects [9,10]. Paeoniflorins contain a pinene skeleton derived from the geranyl cation of the monoterpenoid precursor (E)-geranyl diphosphate (GPP). Class I terpene synthases catalyze the cyclization of the geranyl cation, forming α-terpinyl carbocation, which undergoes further cyclization to produce pinyl carbocation, and eventually α- or β-pinene through deprotonation [11]. These molecules are often further modified by cytochromes P450 (CYPs) and UDP-glycosyltransferase, resulting in a variety of structures [12,13,14].

The biosynthesis of terpenoids in *P. lactiflora* plays a crucial role in its pharmacological activity. Terpenoids, synthesized via various terpenoid synthases, are critical components in the formation of paeoniflorin, a major bioactive compound in *P. lactiflora*. Paeoniflorin exhibits anti-inflammatory, antioxidant, and neuroprotective properties, which are attributed to the complex biochemical pathways involving monoterpene and sesquiterpene synthases [15]. The elucidation of these biosynthetic pathways allows for a deeper understanding of how terpenoids contribute to the therapeutic effects of *P. lactiflora*, including its use in traditional Chinese medicine for treating ailments such as pain, inflammation, and cardiovascular diseases [16].

In other medicinal plants, the study of terpenoid synthases has revealed similar pharmacological relevance. For instance, *Salvia miltiorrhiza,* a well-known medicinal plant, produces tanshinones, diterpenoids that are synthesized via specific diterpene synthases and are recognized for their cardiovascular and anticancer properties [17]. Similarly, *Artemisia annua* contains sesquiterpene artemisinin, whose biosynthesis involves sesquiterpene synthases, making it a key compound in the treatment of malaria [18]. These examples demonstrate how the biosynthetic pathways of terpenoids, driven by terpenoid synthases, are directly linked to the medicinal properties of plants, providing a foundation for the development of pharmaceuticals and bioactive compounds [18]. Future research on *P. lactiflora* and other medicinal plants can explore genetic and metabolic engineering approaches to optimize terpenoid biosynthesis, enhancing their pharmacological applications.

Terpene synthases (TPSs) are encoded by large TPS gene families, classified into seven major subfamilies (TPS-a to TPS-h). These enzymes produced the diverse cyclic and acyclic terpene core structures found in plants [19]. TPS-a, TPS-b, and TPS-g are angiosperm-specific subfamilies, while the TPS-e/f subfamily is present in angiosperms and gymnosperms [20]. TPS-c exists in land plants. TPS-d is a gymnosperm-specific subfamily, and the TPS-h subfamily only appears in *Selaginella moellendorffii* [21]. Most plant TPSs have conserved motifs; the N-terminal domain contains the R(R)X8W motif (R for arginine, W for tryptophan, and X for any amino acid), and the C-terminal domain has the DDXXD and NSE/DTE motifs, which flank the entrance of the active site and function in binding to a trinuclear magnesium cluster [22,23]. The DDXXD motif coordinates divalent ions, and water molecules, and stabilizes the active site [24,25].

The novel TPSs identified in this study demonstrate both structural and functional diversity, expanding the known repertoire of terpenoid biosynthetic enzymes in *P. lactiflora*. The identification of these previously uncharacterized TPSs offers significant insights into the biosynthesis of terpenoids, which are critical in the production of pharmacologically important compounds [26]. Furthermore, the discovery of TPSs producing α-pinene and other monoterpenes may provide new opportunities for the metabolic engineering of *P. lactiflora* to enhance the production of medically relevant terpenoids, including paeoniflorin and related compounds [27]. Additionally, sesquiterpenes identified through the sesquiterpene synthases also hold potential for further pharmacological exploration, as sesquiterpenes are well-known for their anti-inflammatory and anti-cancer properties [28]. In conclusion, our findings add new dimensions to the understanding of terpenoid biosynthesis in *P. lactiflora*, with implications for both basic science and medicinal applications.

## 2. Results

### 2.1. Metabolomic Analysis of P. lactiflora

**Figure 1:**
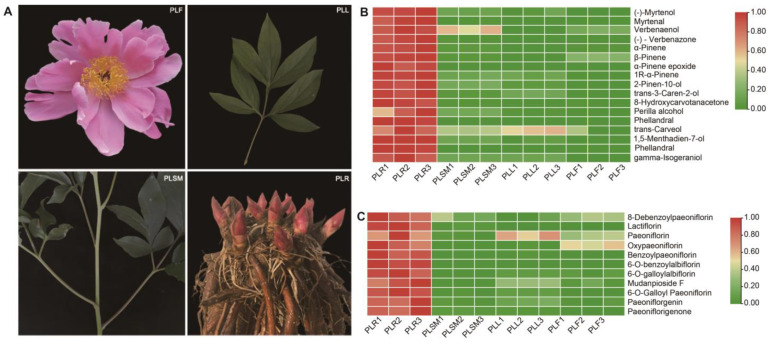
Metabolome results for different tissues of *P. lactiflora*. (**A**): different tissue parts of *P. lactiflora*. (**B**): Cluster analysis of terpenoids in volatile compounds across different tissue parts of *P. lactiflora*.; (**C**): Heatmap analysis of monoterpenoids in different tissue parts of *P. lactiflora*. The color gradient represents the intensity of metabolite expression, with red indicating higher expression levels and blue indicating lower expression levels. Note: PLR: root; PLSM: stem; PLL: leaf; PLF: flower; and Three biological replicates per tissue site.

Metabolomic analysis of *P. lactiflora* has identified over 180 compounds across various species of the genus *Paeonia* [9]. Here, we conducted metabolomics analysis on various tissues (root, stem, leaf and flower) of *P. lactiflora* plants and identified metabolites with significantly different accumulation patterns. We detected 212 compounds. Among these, 36 terpenoids were detected. A total of 46 terpenoids were detected, including 36 monoterpenes, five sesquiterpenes, and five diterpenes (Figure 1B and Figure S1). Monoterpenes primarily accumulate in roots and include bicyclic structures (such as α-pinene and β-pinene), monocyclic structures (such as γ-terpinene), and linear structures (such as 4-hexen-1-ol). Sesquiterpenes consist of linear structures (such as nerolidol), bicyclic structures (such as drimenin), and macrocyclic structures (such as iso-caryophyllene).

Terpenoid profiles were analyzed using UPLC-ESI-Q TRAP-MS/MS with the MWDB system, detecting 54 compounds. This method allows for the qualitative and quantitative analysis of thousands of compounds simultaneously. Results showed that monoterpenoids primarily accumulated in the roots (Figure 1C). α-Pinene was exclusively found in the roots. The accumulation patterns of pinene-type monoterpenes were similar to those of paeoniflorins, indicating that the biosynthesis of paeoniflorins mainly occurs in the roots.

### 2.2. Monoterpenoid and Sesquiterpenoid Biosynthesis Network in P. lactiflora

**Figure 2:**
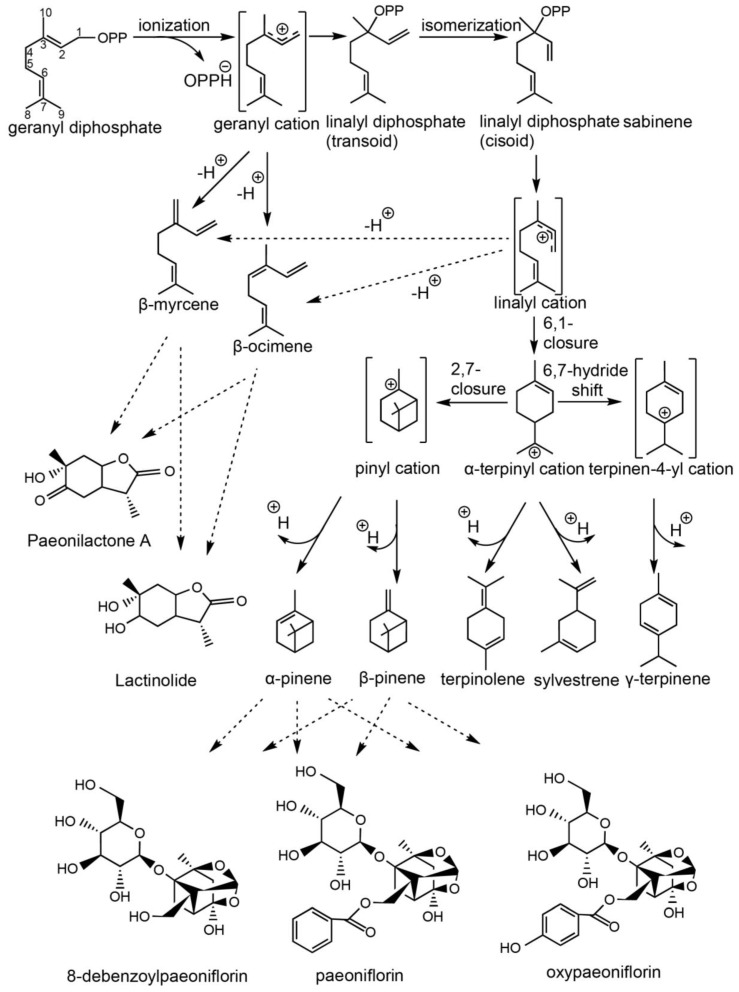
The reaction mechanisms of all monoterpene synthases start with the ionization of the geranyl diphosphate substrate. The numbering of carbon (1–10) atoms of intermediates and products refers to that for GPP. The resulting carbocation can undergo a range of cyclizations, hydride shifts and rearrangements before reaction is terminated by deprotonation or water capture. The formation of cyclic monoterpenes requires the preliminary isomerization of the geranyl cation to a linalyl intermediate capable of cyclization. The production of the initial cyclic species, the a-terpinyl cation, opens the door to secondary cyclizations. The reaction pathways to the major products of all cloned monoterpene synthases are shown. The formation of the acyclic monoterpenes β-myrcene and β-ocimene might proceed either via the geranyl cation or via the linalyl cation. All generated monoterpene compounds may be prerequisite substances for paeoniflorin or albiflorin, indicated by dashed lines.

Terpenoids exhibit remarkable structural diversity, characterized by various carbon skeletons and a broad large of functional groups. This complexity has long fascinated natural products chemists for years, prompting investigation into their biosynthetic origins. Based on metabolome analysis and prior research, we examined the reaction mechanisms of all monoterpene synthases in *P. lactiflora* (Figure 2). The carbocation intermediates generated can undergo multiple cyclizations, hydride shifts and rearrangements before finalizing the reaction through deprotonation or water capture. The formation of cyclic monoterpenes requires the initial isomerization of the geranyl cation to a linalyl intermediate, which then cyclizes. The creation of the α-terpinyl cation facilitates subsequent secondary cyclizations. The pathway leading to the central intermediate is highlighted in grey. The reaction mechanisms to the major products of all cloned monoterpene synthases are illustrated. Acyclic monoterpenes, such as β-myrcene and β-ocimene, may be produced either through the geranyl cation or via the linalyl cation.

**Figure 3:**
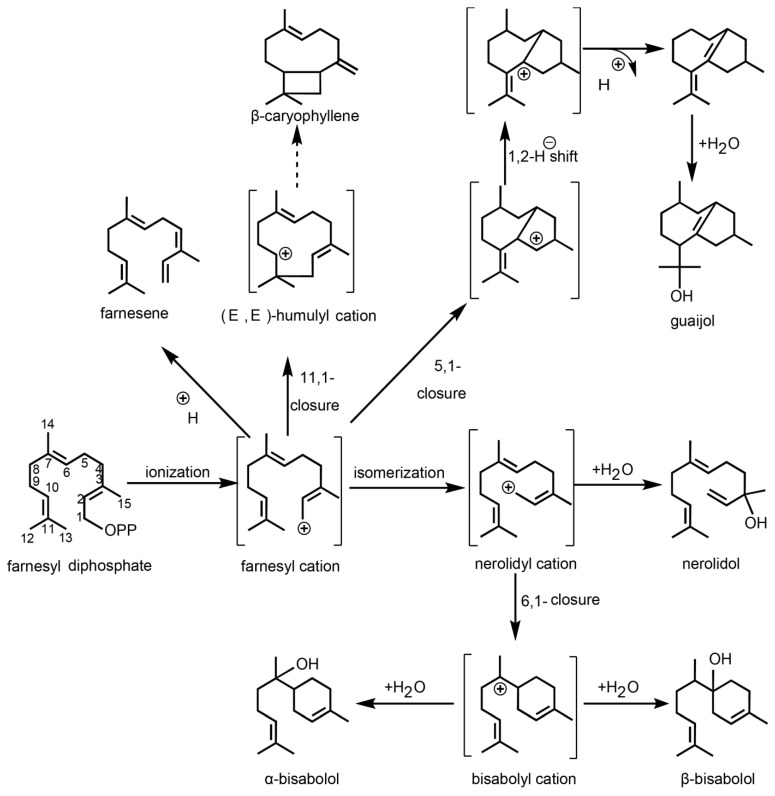
The reaction mechanism of all sesquiterpene synthases start with the ionization of FPP. The numbering of carbon (1–15) atoms of intermediates and products refers to that for FPP. The resulting carbon cation undergoes a range of cyclizations, some of which are preceded by isomerization to a nerolidyl intermediate.

The formation of sesquiterpenes from farnesyl diphosphate (FPP) catalyzed by sesquiterpene synthases, involves carbocation-based reaction mechanisms similar to those observed in monoterpene synthases. However, the larger carbon skeleton of FPP and its three double bonds, as opposed to two, greatly enhance the structural diversity of the resulting products. Based on metabolomic analysis and prior studies, we outline the key steps in these mechanisms in *P. lactiflora*. The resulting carbocation undergoes a series of cyclizations, often preceded by isomerization to a nerolidyl intermediate (Figure 3). Subsequent steps include secondary cyclization, deprotonation to a form neutral intermediate, hydride and methyl shifts, and Wagner–Meerwein rearrangements. The reaction mechanism progresses via the neutral intermediate germacrene A, which is re-protonated to produce bicyclic sesquiterpenes.

### 2.3. Identification of Multiple TPSs in an P. lactiflora Transcriptome

**Figure 4:**
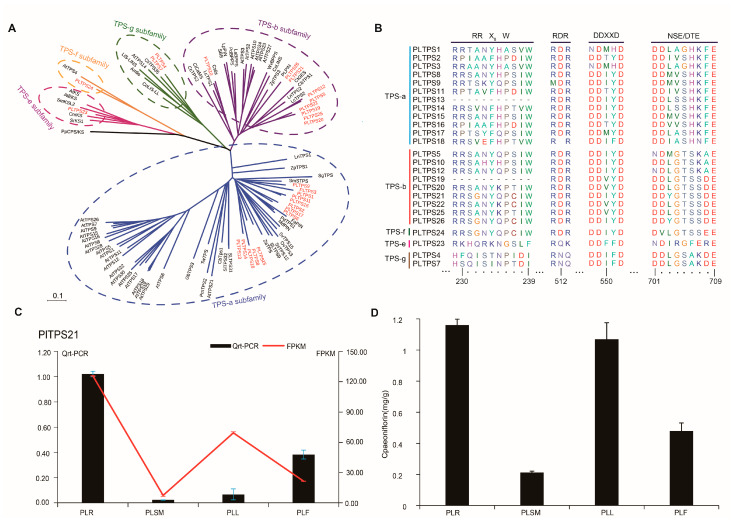
Transcriptome results for different tissues of *P. lactiflora*. (**A**), Phylogenetic tree of PlTPSs. The tree was established using the Neighbor-Joining method (Bootstrap Replications = 1000). The tree was established using the Neighbor-Joining method (Bootstrap Replications = 1000), sequences were selected from GenBank based on their authentication in the literature (unless otherwise indic ated). The information of the TPSs is shown in Table S5. (**B**), Multiple sequence alignment of PlTPSs predicted amino acid sequences of *P. lactiflora*. (**C**), Relative expression level of *P. lactiflora* of PlTPS21 by RT-qPCR and the reads per kilobase million value from the *P. lactiflora* transcriptome. Among them, the horizontal axis represents different tissue parts, PLR: root, PLSM: stem, PLL: leaf, PLF: flower; The left y-axis represents the Qrt PCR results, and the right y-axis represents the transcriptome FPKM values. The error bars show the SDs from the mean value (n = 3), statistically significant based on a Student’s *t*-test (*p* < 0.05). (**D**), Content of paeoniflorin in different tissue parts of *P. lactiflora* by UPLC-MS. (the error bars show the SDs from the mean value (n = 3), Statistically significant based on a Student’s *t*-test (*p* < 0.05).

To investigate the molecular pathways involved in monoterpenoids production, we constructed an expression atlas and examined tissues-specific expression changes in *P. lactiflora* across root, stem, leaf, and flower. A total of 147709 transcripts were assembled, with an average length of 761 bp, and an N50 length of 1217 bp (Table S1). (Accession number: CRA017941). Homology searches and functional annotation of the *P. lactiflora* transcriptome identified 32 terpene synthases (TPSs), of which 26 had full-length ORFs. To elucidate the physiological roles of these candidate TPSs, we analyzed their transcript levels in various organs using FPKM (Fragments Per Kilobase of transcript per Million fragments mapped) values (Figure 4C). The analysis revealed that seven PlTPSs exhibited higher expression in leaves, 12 in flowers, and seven were specifically expressed in roots. qRT-PCR was used to verify these expression profiles, confirming consistency with the transcriptome data, particularly for PlTPS21 (Figure 4C and Figure S2). The qRT-PCR expression profiles were consistent with the transcriptome data, particularly for PlTPS21 (Figure 4C and Figure S2). Phylogenetic analysis of 26 PlTPSs, compared with a representative set of TPSs, identified 25 as class I TPSs based on conserved DDxxD and NSE/DTE motifs (Figure 4B). PlTPS23, which contains the DxDD motif, was tentatively classified as a class II diTPS, clustering within the TPS-e subfamily with known kaurene synthases of *Arabidopsis thaliana* (AtEKS). PlTPS24 was classified within the TPS-f subfamily, commonly associated with homoterpene biosynthesis [29]. Phylogenetic analysis of 24 TPS genes, including monoterpene and sesquiterpene synthases, revealed their distribution across different subfamilies (Figure 4A). The TPS-a subfamily contains 13 genes likely involved in sesquiterpene biosynthesis, TPS-b contains nine genes likely functioning as monoterpene synthases, and TPS-g contains two genes potentially functioning as both monoterpene and sesquiterpene synthases.

### 2.4. Functional Characterization of Putative PlTPSs

To assess the enzymatic activities of PlTPS1-26 and determine if the previously identified PlPIN exhibits α-pinene synthase activity, we conducted in vitro assays using yeast expression systems. PlTPSs were expressed in *Saccharomyces cerevisiae* strains SP-G16 (producing GPP) or YH-C15 (producing FPP) using the pESC-Trp yeast expression vectors. The products were analyzed by GC–MS and identified by comparison with chemical standard or matched with the NIST mass spectral library for unmatched products (Figures S3 and S4). Four enzymes (TPS5, TPS8, TPS21 and TPS22) were identified as mono-TPSs, while five PlTPSs (PlTPS4, PlTPS6, PlTPS17, PlTPS22 and PlTPS24) were identified as sesqui-TPSs.

**Figure 5:**
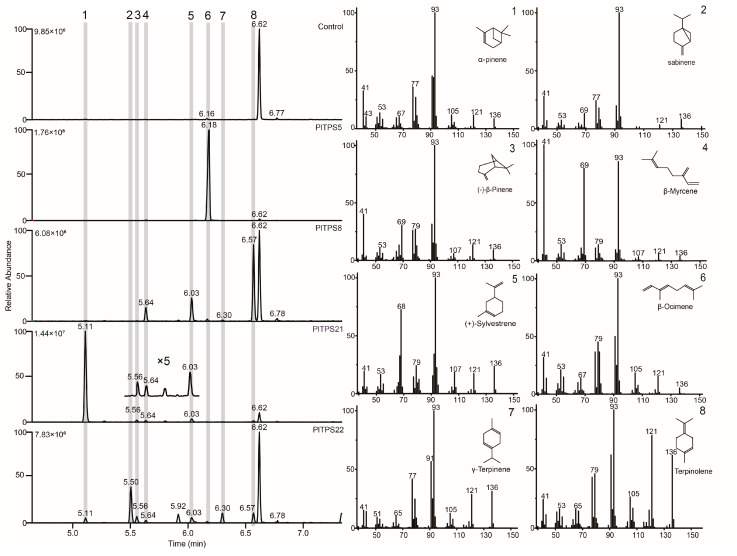
GC–MS analysis of *P. lactiflora* Class I terpene synthase in vivo assays with engineered yeast (SP-G16). A, Extracted ion chromatograms (EICs) of *m*/*z* 121 + 136 of four PlTPSs (PlTPS5, PlTPS8, PlTPS21 and PlTPS22). The enzymes with pESC-Trp was used as controls. Mass spectrograms (EI) of reaction products (1–8) correspond one-to-one with structure of products (1–8).

In assays with SP-G16 (Figure 5), PlTPS5 produced a single product, β-ocimene, indicating it is a monofunctional TPS. PlTPS8, PlTPS21, and PlTPS22 produced multiple products, suggesting versatility. PlTPS21 primarily produced α-pinene along with minor products β-pinene 3, β-myrcene 4, and sylvestrene 5. PlTPS8 generated terpinolene as the major product and three minor products—β-myrcene 4, sylvestrene 5, and γ-terpinene 7. PlTPS22 produced all seven products identified for PlTPS8 and PlTPS21.

**Figure 6:**
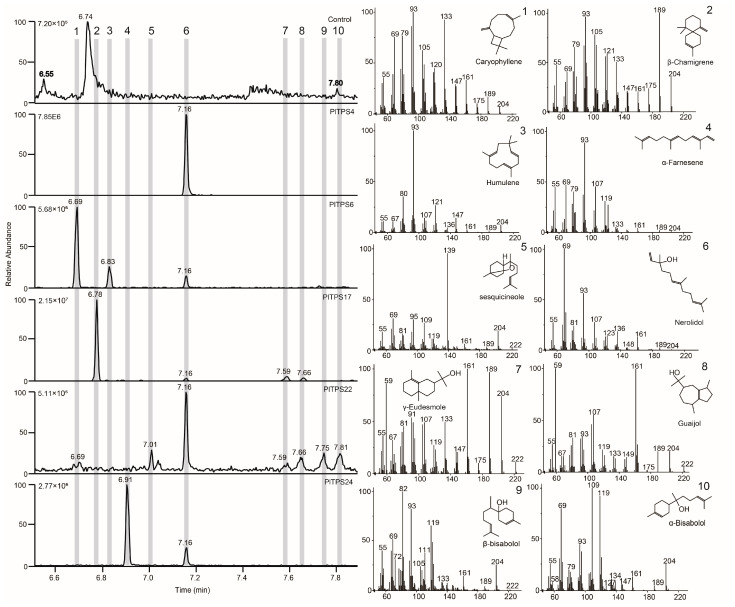
GC–MS analysis of *P. lactiflora* Class I terpene synthase in vivo assays with engineered yeast that can produce FPP. A, Extracted ion chromatograms (EICs) of *m*/*z* 189 of five PlTPSs (PlTPS4, PlTPS6, PlTPS20, PlTPS22, and PlTPS24). The enzymes with pESC-Trp was used as controls. Mass spectrograms (EI) of reaction products (1–10) correspond one-to-one with structure of products (1–10).

In assays with YH-C15 (Figure 6), nerolidol was detected in all five sesquiterpene TPSs. PlTPS4 exclusively produced nerolidol 6, while PlTPS22 was a major producer with six minor products. PlTPS24 also produced nerolidol 6 but as a minor product, with α-farnesene 4 as the major product. PlTPS6, PlTPS17, PlTPS22, and PlTPS24 produced multiple products as versatile sesquiterpene TPSs. Notably, PlTPS6 produced caryophyllene 1 as the major product along with minor products humulene 3 and nerolidol 6. PlTPS17 produced β-chamigrene 2 as the major product, with minor products nerolidol 6, γ-eudesmol 7, and guaijol 8.

Based on these results, PlTPS5 may be involved in the synthesis of linear monoterpenes, while PlTPS21 mainly produces the bicyclic monoterpene α-pinene, a crucial precursor in the biosynthesis of paeoniflorin, the main active compound in *P. lactiflora*. Thus, PlTPS21 may play a key role in paeoniflorin biosynthesis. PlTPS8 primarily produces monocyclic monoterpenes, indicating its role in their synthesis. Among sesquiterpenes, PlTPS4, PlTPS22, and PlTPS24 primarily produce linear sesquiterpenes (including α-farnesene and nerolidol), suggesting their involvement in linear sesquiterpene biosynthesis. Conversely, PlTPS6 and PlTPS17 mainly produce bicyclic sesquiterpenes, indicating their role in the biosynthesis of these structures.

## 3. Discussion

*P. lactiflora* Pall. contains over 180 compounds, including terpenes, phenols, flavonoids, essential oils, and tannins, with monoterpenoids and monoterpenoid glycosides as predominant constituents. Despite their abundance, the molecular mechanisms behind terpenoid biosynthesis in *P. lactiflora* are largely unknown. This study identified and characterized 26 full-length terpene synthases (PlTPSs) from *P. lactiflora*. Among these, four mono-TPSs and five sesqui-TPSs were functionally active in vivo, including the previously reported α-pinene synthase (PlPIN). Among these, one of the monoterpene synthases was found to produce α-pinene, a compound that is likely the precursor to the paeoniflorin backbone, a major bioactive constituent in *P. lactiflora*. This finding aligns with previous studies where α-pinene was identified as a precursor in monoterpene-based bioactive compounds in other medicinal plants such as *Salvia miltiorrhiza* and *Artemisia annua* [29,30]. However, this study provides new insights specific to *P. lactiflora* by identifying α-pinene as a potential biosynthetic intermediate leading to paeoniflorin, further supporting its medicinal role.

Phylogenetic analysis revealed that most PlTPSs cluster with the TPS-a and TPS-b subfamilies, associated with monoterpene or sesquiterpene functions. Notably, PlTPS24 grouped with AtTPS4 in the TPS-f subfamily, suggesting it might be a multifunctional terpenoid synthase, contrary to previous studies. This finding warrants further investigation, possibly through in vitro expression studies. Gene expression and metabolite analyses linked PlTPS21 with the distribution of paeoniflorins-monoterpenoids primarily found in roots—supporting its role in paeoniflorin biosynthesis in *P. lactiflora*. Homoterpenes in *P. lactiflora* often participate in tritrophic plant-insect herbivore-predator/parasitoid interactions [31,32,33].

Despite these promising findings, several limitations should be acknowledged. The specificity of the expression system used may affect the accurate characterization of TPS activity. For instance, heterologous expression in microbial systems might not fully reflect the natural environment of the plant cell, potentially missing post-translational modifications or interactions that could influence TPS functionality [34]. Moreover, while we identified nine TPS genes, it is likely that additional, yet undiscovered TPSs exist within *P. lactiflora*. The complexity of the terpenoid biosynthesis pathways suggests that further investigation, potentially through a more comprehensive transcriptomic or genomic analysis, may reveal additional TPS genes involved in the biosynthesis of terpenoids with medicinal importance [35,36].

In conclusion, our findings add new dimensions to the understanding of terpenoid biosynthesis in *P. lactiflora*, with implications for both basic science and medicinal applications. The newly identified TPSs not only contribute to the diversity of terpenoid compounds produced by the plant but also offer potential targets for metabolic engineering to optimize the production of therapeutically valuable terpenoids. Additionally, comparative studies across other medicinal plants could offer insights into the evolutionary conservation and diversification of TPSs. Future research should also explore the regulatory mechanisms governing TPS expression and their interaction with other biosynthetic pathways.

## 4. Materials and Methods

### 4.1. Plant Materials

The *P. lactiflora* Pall. plant materials used in this study (two years old) were all grown in the same public planting base in Heze, Shang Dong Province; plants were obtained in June 2021. Four tissues of each plant’s roots, stems, flowers and leaves were examined during the *P. lactiflora* flowering period (Figure 1A). All the collected tissues were quickly placed in liquid nitrogen and stored at −80 °C before RNA extraction.

### 4.2. Total RNA Isolation and cDNA Synthesis

Total RNA was extracted using an RNA isolation kit (HuaYueYang Biotechnology, Beijing, China) following the manufacturer’s instructions. The integrity and concentration of total RNA were monitored using 1.0% agarose gel electrophoresis and a NanoDrop ND-1000 (NanoDrop Technologies, Wilmington, DE, USA). To assess the quality of the extracted RNA, we used an Agilent Bioanalyzer 2100 system (Agilent Technologies, Santa Clara, CA, USA). Use PrimeScript™ RT reagent kit with gDNA Eraser (Takara Bio., San Jose, CA, USA) to synthesize cDNA with 1 µg total RNA.

### 4.3. Transcriptome Sequencing, De Novo Assembly and Annotation

RNA was prepared for sequencing using Illumina TruSeq sample preparation kit v2 (Illumina San Diego, San Diego, CA, USA). The fragments were sequenced with paired ends (2 × 150 bp) on a Illumina HiSeq 2500 platform (Illumina San Diego, San Diego, CA, USA) by the Annoroad Gene Tech. (Beijing, China) Co., Ltd. Adapter sequences were removed from raw reads and reads were trimmed at the ends to Q30, using the fastq-mcf tool from ea-utils (Analysis/ea-utils). Processed reads were de novo assembled and estimated using the Trinity (Trinity Release v2.4.0) pipeline according to guidelines in the published protocols. ORFs were predicted by TransDecoder. Trinotate was used for the functional annotation of unigenes and ORFs to known sequence data (BLAST+/SwissProt), protein domain identification (HMMER/PFAM), protein signal peptide and transmembrane domain prediction (singalP/tmHMM), and comparison to currently current annotation databases (EMBL Uniprot eggNOG/GO Pathways databases). In order for the number of fragments to truly reflect the expression level of the transcript, it is necessary to normalize the number of MappedReads in the sample and the transcript length. FPKM (Fragments Per Kilobase of transcript per Million fragments mapped) is used as the index to measure transcript or gene expression level, FPKM is calculated with the following formula:

*FPKM* = *mapped fragments of transcript*/(*Total Count of mapped frangments* (*Millions*) × *Length of transcript* (*kb*)) (1)

### 4.4. Metabolite Analysis of P. lactiflora by UPLC-ESI-Q TRAP-MS/MS

For metabolite analyses, the *P. lactiflora* tissues (e.g., root, stem, leaf, flower, and seeds with six biological replicates) were ultrasound extracted with methanol at 25 °C for 1 h, and then centrifuged at 12,000× *g* for 15 min at room temperature. The supernatant was directly analyzed by UPLC-MS/MS. Ultrahigh-performance liquid chromatography (UPLC) (SHIMADZU Nexera X2, https://www.shimadzu.com.cn/ accessed on 27 September 2024) and tandem mass spectrometry (tandem mass spectrometry, MS/MS) (Applied Biosystems 4500 QTRAP, Framingham, MA, USA http://www.appliedbiosystems.com/ accessed on 27 September 2024) were used to collect terpenoid metabolite data. Based on the self-established Metware Database (MWDB), the terpenoid metabolites were qualified according to the secondary spectrum information. Triple quadrupole (QQQ) scans were acquired on a triple quadrupole mass spectrometer (Q TRAP), AB4500 Q TRAP UPLC/MS/MS System, equipped with an ESI Turbo Ion-Spray interface, operating in positive and negative ion mode and controlled by Analyst 1.6.3 software (AB Sciex, Framingham, MA, USA) [37,38]. The column temperature was set at 40 °C. The flow rate was kept at 350 μL·min^−1^. Mobile phases were water (with 0.1% Formic acid) (A) and acetonitrile (with 0.1% Formic acid) (B). The source temperature was set at 550 °C. The IonSpray voltage was set at +5.5KV (positive ion mode) and −4.5 KV (negative ion mode). The Ion Source Gas (GS) was set at 50 psi [39] and 60 psi (GSII), and the curtain gas [39] was set at 25 psi.

The volatile metabolites were detected based on the GC-MS detection platform and self-built database, and volatile metabolites were qualitatively and quantitatively analyzed via mass spectrometry-based on the MWGC database. MassHunter quantitative software B.09.00.was used to process the sample mass spectrometry files, and quantitative ions were selected to integrate and correct chromatographic peaks to increase the accuracy of quantification. Qualitative analysis was performed with Workflows B software 08.00 open the browse off the plane after the mass spectrometry analysis of original data and used for qualitative analysis. The samples (1.0 g each) were placed in a headspace bottle and were extracted using SPME with a 120 μm DVB/CAR/PDMS fiber head and kept in a 100 °C bath for 15 min. The injection temperature was 250 °C and the initial temperature was 40 °C, ion trap heating, 250 °C, He-lium (99.999%) 1.2 mL/min, splitless injection. The interface temperature was 250 °C, ion source temperature was 230 °C, Quadrupole temperature was 150 °C and electron bombardment source was 70 eV. The oven program was 40 °C for 3.5 min, ramped at rate 10 °C min^−1^ to 100 °C, ramped at rate 7 °C min^−1^ to 180 °C, ramped at rate 25 °C min^−1^ to 280 °C and held for 5 min. The scanning range of mass spectrometry was *m*/*z* 50~550.

### 4.5. Content of Paeoniflorin in Different Tissue of P. lactiflora by UPLC-MS

Powdered samples (0.5 g each) from different tissue parts of *Paeonia lactiflora* (root, stem, leaf, and flower) were treated with 40% methanol solution in a 50 mL stoppered bottle. The samples were subjected to ultrasonic treatment (240W power, 45 kHz frequency) for 30 min, then cooled. The weight loss was compensated by adding 40% methanol solution, and the final extracts were filtered through a 0.20 μm membrane.

The samples were analyzed using a Thermo Q Exactive™ Plus LC-MSsystem (Thermo, Waltham, MA, USA) with a Waters CSH™ C18 (Milford, MA, USA, 100 mm × 2.1 mm I.D., 1.7 µm) for chromatographic separation. The mobile phase was consisted of solvent A, pure water with 0.1% formic acid and solvent B was acetonitrile. Sample measurements were performed with a gradient program that employed the starting conditions of 95% A, 5% B. Within 12 min, a linear gradient to 70% A, 30% B was programmed and kept for 6 min, and a composition of 100% B was kept for 6 min. Subsequently, a composition of 95% A, 5.0% B was adjusted within 0.2 min and kept for 2.8 min. Column temperature was maintained at 30 °C, with detection by a diode array detector (DAD) wavelength set at a wavelength range of 210–400 nm. MS parameters were sheath gas, N_2_ (45 arb); nebulizing gas, N_2_ (10 arb); capillary voltage, 3.8 kV; stepped normalized collisional energy, 30 eV/50 eV/70 eV; data acquisition rate, 1 spectrum/s. The scan ranges were defined as *m*/*z* 150–1250 and *m*/*z* 50–1250 for MS acquisition.

Using the concentrations of the standard paeoniflorin solutions (0.2 mg/mL, 0.1 mg/mL, 0.05 mg/mL, 0.02 mg/mL, 0.01 mg/mL, 0.005 mg/mL, and 0.002 mg/mL), we plotted a standard curve with the paeoniflorin concentration on the x-axis and the corresponding the area under the curve on the y-axis. Fit the data to a linear regression model to establish the relationship between concentration and peak area (AUC). The equation of the standard curve will typically take the form:

Y = mX + b(2)

where Y is the peak area, X is the concentration, m is the slope, and b is the y-intercept.

### 4.6. PlTPSs Screening and Bioinformatic Analysis

The genes were filtered according to their transcriptome annotation information to comprehensively identify TPS genes in *P. lactiflora*. The nucleotide and deduced amino acid sequences were analyzed, and the BLASTP tool on the NCBI online database (http://www.ncbi.nlm.nih.gov/ (accessed on 27 September 2024)) was used to compare the sequences. Multiple sequence alignment was implemented by ClustalW software 2.1., and MEGA 7.0 software was used to construct a phylogenetic tree using the NJ (neighbor-joining) method (bootstrap = 1000). TPS genes were subsequently amplified with specific primers (Supplementary Materials Tables S2 and S3) and cloned and inserted into pESC-Trp with a seamless cloning kit (TransGen Biotech, Beijing, China).

### 4.7. Isolation and Cloning of PlTPSs Coding Sequences

The specific primers were designed to amplify the PlTPSs ORFs according to the RNA-seq information (all primers were in the Tables S2 and S3). The PCR products were purified and cloned into the T-Vector (pEASY-Blunt Zero Simple Vector, TransGen, Beijing, China). The rebulide plasmids were isolated by Kit, and sequenced by Sangon Biotech (Shanghai) Co., Ltd. (Shanghai, China). Amplicons were digested with Bamh I restriction enzymes (New England Biolab, Ipswich, MA, USA) and inserted into the expression vector pESC-Trp with a pEASY-Uni Seamless Cloning and Assembly Kit (TransGen, Beijing, China).

### 4.8. Functional Characterization of PlTPSs in Yeast

The ORF regions of PlTPSs were subcloned into pESC-Trp digestion with PCR amplification with a pEASY-Uni Seamless Cloning and Assembly Kit, amplicons were digested with BamHI restriction enzymes. The constructed vectors pESC-Trp::PlTPSs were transformed into the yeast strain (SP-G16) of producing GPP (CEN.PK2-1D MAT α; ura3-52, trp1-289, leu2-3, 112, his3Δ1, MAL2-8C, SUC2, XI-5::P_TEF1_-Cas9-T_CYC1t_, Gal80Δ::P_GAL1_-ERG10-T_TPI1_-P_GAL10_-ERG13-T_PGI_, Ypl062wΔ::P_GAL1_-tHMG1-T_ADH1_-P_GAL10_-ERG12-T_PDC1_, XI-3::P_GAL1_-ERG8-T_RPS2_-P_GAL10_-ERG19-T_TDH1_, Rox1Δ::P_GAL1_-IDI1-T_CCW12_-P_GAL10_-ERG20^F96W-N127W^-T_RPL9A_, YPRCΔ15::P_GAL1_-tHMG1-T_ADH1_-P_GAL10_-EfmvaE-T_PDC1_, pERG7), or into the yeast strain (YH-C15) of producing FPP (CEN.PK2-1D, MAT α; ura3-52, trp1-289, leu2-3,112, his3Δ1,MAL2-8C, SUC2, XI-5::P_TEF1-_Cas9-T_CYC1t_, Gal80Δ::P_GAL1_-ERG10-T_TPI1_-P_GAL10_-ERG13-T_PGI_, Ypl062wΔ::P_GAL1_-tHMG1-T_ADH1_-P_GAL10_-ERG12-T_PDC1_, XI-3::P_GAL1_-ERG8-T_RPS2_-P_GAL10_-ERG19-T_TDH1_, Rox1Δ::P_GAL1_-IDI1-T_CCW12_-P_GAL10_-ERG20-T_RPL9A_, YPRCΔ15::P_GAL1_-tHMG1-T_ADH1_-P_GAL10_-EfmvaE-T_PDC1_, pERG1). The yeast cells harboring the expression vector were selected on SD-Trp media and grown at 30 °C, 220 r. min^−1^ overnight activation culture for 3 days in an incubator. And extracted thrice using an equal volume of ethyl acetate, and the extracts were dried with N_2_ and dissolved in 250 µL of ethyl acetate for GC-MS analysis.

### 4.9. The Products of PlTPSs Analysis by GC-MS

The GC-MS analysis was performed by a Trace 1310 instrument equipped with a TSQ 8000 mass detector and then separated by a TG-5 MS column (30 m × 0.25 mm I.D., DF = 0.25 μm; Thermo Scientific, Waltham, MA, USA) in electron ionization mode was used for GC-MS analysis. Helium was used as a carrier gas at a flow rate of 1.0 mL^−1^·min and the temperature program was as follows: initial column oven temperature of 50 °C for 2 min, an increase of 40 °C min^−1^ to 210 °C, an increase of 5 °C min^−1^ to 250 °C, an increase of 40 °C to 300 °C and holding for 5 min. The ion trap temperature was 280 °C. We determined the final product by comparing the retention time and mass spectrometry data with previously characterized enzyme products or standards.In the end, we used our built-in database for retrieval and result confirmation.

### 4.10. Quantitative Real-Time PCR Analysis

Total RNA was extracted from *P. lactiflora* tissues using a Quick RNA Isolation Kit (HuaYueYang Biotechnology, Beijing, China) following the manufacturer’s instructions. First-strand cDNA was synthesized using the PrimeScript™ RT Reagent Kit with gDNA Eraser (Takara Bio., Kusatsu, Shiga, Japan). Relative transcript abundance was determined by qRT-PCR using the A SYBR Green kit (TaKaRa Corp., Kusatsu, Shiga, Japan) on a Roche LightCycler480 (Roche, Basel, Switzerland). The primers used for qRT-PCR analysis are listed in Table S4. The gene for actin was used as the endogenous control. At least three independent experiments were performed for each analysis. Primer specificity was assessed by melting curve analysis. The results were normalized to the housekeeping gene Actin, and relative expression levels were calculated as the mean of three technical replicates of three biological replicates.

## 5. Conclusions

In this paper, transcriptome sequencing and metabolome analysis were conducted on the roots, stems, leaves and flowers of *P. lactiflora*. A total of 32 PlTPS were identified. Phylogenetic analysis revealed that all PlTPSs were primarily clustered in the TPS-a, TPS-b and TPS-g subfamilies, and eight functional PlTPSs were identified in vitro. Among them, PlTPS5, PlTPS8, PlTPS21 and PlTPS22 can produce eight monoterpenoids, while PlTPS4, PlTPS6, PlTPS17, PlTPS22 and PlTPS24 can produce ten different sesquiterpenoids, with TPS22 being a bifunctional gene. This study lays a molecular biology foundation for the biosynthesis of paeoniflorin.

## Data Availability

The data presented in this study are openly available in GSA at https://ngdc.cncb.ac.cn/gsub/submit/gsa/subCRA028870 (accessed on 27 September 2024).

## Supplementary Materials

The following supporting information can be downloaded at: https://www.mdpi.com/article/10.3390/molecules29194662/s1, Table S1: Transcriptome assembly results of P. lactiflora; Table S2: Primers for cloning the ORFs of PlOSCs; Table S3: Primers for vector construction of PlTPSs; Table S4: Primers for Qrt-PCR of PlTPS21; Table S5: The information of TPSs from representative characterized subfamily genes; Figure S1: Metabolome results for different tissues of P. lactiflora; Figure S2: Relative expression level of P. lactiflora of PlTPSs by RT-qPCR and the reads per kilobase million value from the P. lactiflora transcriptome; Figure S3: GC–MS analysis of P. lactiflora Class I terpene synthase in vitro assays with engineered yeast that can produce GPP. The m/z of products were matched with NIST mass spectral library for identification of a potential terpenes.; Figure S4: GC–MS analysis of P. lactiflora Class I terpene synthase in vitro assays with engineered yeast that can produce FPP. The m/z of products were matched with NIST mass spectral library for identification of a potential terpenes.
