# Supplementary material for: Functional Identification of the Terpene Synthase Family Involved in Biosynthesis in Paeonia lactiflora
Source: Molecules. 2024 Sep 30;29(19):4662. doi: 10.3390/molecules29194662 (PMC11478036; doi:10.3390/molecules29194662)
Supplement: Supplementary file 1 [file molecules-29-04662-s001.zip › molecules-3193109-supplementary.pdf]

Table S1 Transcriptome assembly results of *P. lactiflora*

Type	Number	Mean Length (bp)	N50
Transcript	147709	761	1217
Unigene	131366	825	1269

Table S2 Primers for cloning the ORFs of PlOSCs

Names	Sequence 5′ to 3
PlTPS1	F: ATGTCTGTTTTAGGTCTGC<br>R: TCAGCAAGGGTTTATGAG
PlTPS2	F:ATGTCTGTCCAAGTCTCACCCG<br>R:TCATACTGGGACAGGCTCTATG
PlTPS3	F:ATGTCCGTTTTAGGTCTGC<br>R:TCAGCAAGGGTTTATGAGC
PlTPS4	F:ATGGCCTATTTCAATACCTC<br>R:CTATAAAGATAAAGTTCCATACAAG
PlTPS5	F:ATGATGAATCTGCGCCTTAC<br>R:TCATTCTTTGCCTGATTTATTTAATAC
PlTPS6	F:ATGTCTCTCCCTGTGTTAGAAAC<br>R:TCATATTGGGATTGGATCAATTAG
PlTPS7	F:ATGGCCTATTTCAATACCTC<br>R:CTATAAAGATAAAGTTCCATACAAG
PlTPS8	F:ATGTCTACTGAAGCTTCCGCTG<br>R:TCATATTGCAAAATGATCAATGAG
PlTPS9	F:CCATGTCTGTCCAAGTCCTGGCTTC<br>R:TCAAGAGGTGTGATCCACTGGAAAAT
PlTPS10	F:ATGGAACTAGTTTCTTTCC<br>R:TTATAGTACATTGATATGTTTACTT
PlTPS11	F:ATGTCTGTCCAAGTCCCAGC<br>R:TCATACTGGCACGGGGTC
PlTPS12	F:ATGAGCCAGTATCTTCAGTGCATAG<br>R:TTAATTAACATTAGTGTTCACCAAAT
PlTPS13	F:ATGTCAATTGGGAAGGAGAAGGCAG<br>R:TCAGCCACACGTGCATAGTTGAG
PlTPS14	F:ATGCAATTAAAGACGGGGC<br>R:TCACATTGCAATAGGATCGG
PlTPS15	F:ATGTCTACTGAAGCTTCCGCTG<br>R:TCATATTGCAATATGATCAATGAG
PlTPS16	F:ATGCAAACCAGCTATTCC<br>R:TCATACTGGGACAGGCTC
PlTPS17	F:ATGTCTTTCCAACTCTCCACCAATG<br>R:TCATATTGGAACAGGATCAATCAGC
PlTPS18	F:ATGTCTGTTCAAGCTGCAACTGGTC<br>R:TCACATTGCGATAGGATCGACCAC
PlTPS19	F:ATGAAGCTTGCCAAAGTGGTGG<br>R:TTAGGGAATGGGCTGAATGAGAAG
PlTPS20	F:ATGGCCATTTGCTCTGTCATTAC<br>R:TTAGGGAATGGGTTGAATGAGAAG
PlTPS21	F:ATGGCTACACGTCTGGTTG<br>R:CTACATGGGAATGGGTTGAAAAAG

PlTPS22	F:ATGTCTATTCATCAGCTTAC<br>R:TTAAGGAATGGGTTGAATAAGTAG
PlTPS23	F:ATGTATCTCTCCAACCCTAGCAACC<br>R:TCATTAAGGACTGCATTTGC
PlTPS24	F:ATGGAATCAACACAAATCACAAT<br>R:TATTTAGAAACAATACCCACTCTTG
PlTPS25	F:ATGAAGAGTTTGACCACGGAC<br>R:TGTTTGACAAAGTTACATTCCTGTG
PlTPS26	F:ATGGCCCTTCAACTTCTCTTC<br>R:GCTAGTTTAGTGAAACAGGTTCCAC

Table S3 Primers for vector construction of PlTPSs

Names	Sequence 5′ to 3
PlTPS1	F: acgtcaaggagaaaaaaccccggatccATGTCTGTTTTAGGTCTGC<br>R: tagtgagtcgtattacggatccTCAGCAAGGGTTTATGAG
PlTPS2	F:acgtcaaggagaaaaaaccccggatccATGTCTGTCCAAGTCTCACCCG<br>R:tagtgagtcgtattacggatccTCATACTGGGACAGGCTCTATG
PlTPS3	F:acgtcaaggagaaaaaaccccggatccATGTCCGTTTTAGGTCTGC<br>R:tagtgagtcgtattacggatccTCAGCAAGGGTTTATGAGC
PlTPS4	F:acgtcaaggagaaaaaaccccggatccATGGCCTATTTCAATACCTC<br>R:tagtgagtcgtattacggatccCTATAAAGATAAAGTTCCATACAAG
PlTPS5	F:acgtcaaggagaaaaaaccccggatccATGATGAATCTGCGCCTTAC<br>R:tagtgagtcgtattacggatccTCATTCTTTGCCTGATTTATTTAATAC
PlTPS6	F:acgtcaaggagaaaaaaccccggatccATGTCTCTCCCTGTGTTAGAAAC<br>R:tagtgagtcgtattacggatccTCATATTGGGATTGGATCAATTAG
PlTPS7	F:acgtcaaggagaaaaaaccccggatccATGGCCTATTTCAATACCTC<br>R:tagtgagtcgtattacggatccCTATAAAGATAAAGTTCCATACAAG
PlTPS8	F:acgtcaaggagaaaaaaccccggatccATGTCTACTGAAGCTTCCGCTG<br>R:tagtgagtcgtattacggatccTCATATTGCAAAATGATCAATGAG
PlTPS9	F:acgtcaaggagaaaaaaccccggatccCCATGTCTGTCCAAGTCCTGGCTTC<br>R:tagtgagtcgtattacggatccTCAAGAGGTGTGATCCACTGGAAAAT
PlTPS10	F:acgtcaaggagaaaaaaccccggatccATGGAACTAGTTTCTTTCC<br>R:tagtgagtcgtattacggatccTTATAGTACATTGATATGTTTACTT
PlTPS11	F:acgtcaaggagaaaaaaccccggatccATGTCTGTCCAAGTCCCAGC<br>R:tagtgagtcgtattacggatccTCATACTGGCACGGGGTC
PlTPS12	F:acgtcaaggagaaaaaaccccggatccATGAGCCAGTATCTTCAGTGCATAG<br>R:tagtgagtcgtattacggatccTTAATTAACATTAGTGTTCACCAAAT
PlTPS13	F:acgtcaaggagaaaaaaccccggatccATGTCAATTGGGAAGGAGAAGGCAG<br>R:tagtgagtcgtattacggatccTCAGCCACACGTGCATAGTTGAG
PlTPS14	F:acgtcaaggagaaaaaaccccggatccATGCAATTAAAGACGGGGC<br>R:tagtgagtcgtattacggatccTCACATTGCAATAGGATCGG
PlTPS15	F:acgtcaaggagaaaaaaccccggatccATGTCTACTGAAGCTTCCGCTG<br>R:tagtgagtcgtattacggatccTCATATTGCAATATGATCAATGAG
PlTPS16	F:acgtcaaggagaaaaaaccccggatccATGCAAACCAGCTATTCC<br>R:tagtgagtcgtattacggatccTCATACTGGGACAGGCTC
PlTPS17	F:acgtcaaggagaaaaaaccccggatccATGTCTTTCCAACTCTCCACCAATG<br>R:tagtgagtcgtattacggatccTCATATTGGAACAGGATCAATCAGC
PlTPS18	F:acgtcaaggagaaaaaaccccggatccATGTCTGTTCAAGCTGCAACTGGTC<br>R:tagtgagtcgtattacggatccTCACATTGCGATAGGATCGACCAC
PlTPS19	F:acgtcaaggagaaaaaaccccggatccATGAAGCTTGCCAAAGTGGTGG<br>R:tagtgagtcgtattacggatccTTAGGGAATGGGCTGAATGAGAAG
PlTPS20	F:acgtcaaggagaaaaaaccccggatccATGGCCATTTGCTCTGTCATTAC<br>R:tagtgagtcgtattacggatccTTAGGGAATGGGTTGAATGAGAAG
PlTPS21	F:acgtcaaggagaaaaaaccccggatccATGGCTACACGTCTGGTTG<br>R:tagtgagtcgtattacggatccCTACATGGGAATGGGTTGAAAAAG

PlTPS22	F:acgtcaaggagaaaaaaccccggatccATGTCTATTCATCAGCTTAC<br>R:tagtgagtcgtattacggatccTTAAGGAATGGGTTGAATAAGTAG
PlTPS23	F:acgtcaaggagaaaaaaccccggatccATGTATCTCTCCAACCCTAGCAACC<br>R:tagtgagtcgtattacggatccTCATTAAGGACTGCATTTGC
PlTPS24	F:acgtcaaggagaaaaaaccccggatccATGGAATCAACACAAATCACAAT<br>R:tagtgagtcgtattacggatccTATTTAGAAACAATACCCACTCTTG
PlTPS25	F:acgtcaaggagaaaaaaccccggatccATGAAGAGTTTGACCACGGAC<br>R:tagtgagtcgtattacggatccTGTTTGACAAAGTTACATTCCTGTG
PlTPS26	F:acgtcaaggagaaaaaaccccggatccATGGCCCTTCAACTTCTCTTC<br>R:tagtgagtcgtattacggatccGCTAGTTTAGTGAAACAGGTTCCAC

Table S4 Primers for Qrt-PCR of PlTPS21

Names	**Sequence 5′ to 3**
Actin	F：AGCTCATCGGTTGAGAAGAACTAC
	R：ACATAGTTGATCCACCACTGAGAAC
PlTPS21	F：GTTTGGGTTACGAGTTTGAGACAG
	R：GCCTCCTCCATGATGCTTTC

Table S5 The information of TPSs from representative characterized subfamily genes

Gene name	Gene Bank NO.	Species	Gene name	Gene Bank NO.
CbTPS1	WKW91725	*Cinnamomum burmanni*	AtTPS1	NP_193322
CoLIS-LL	AFK09265	*Cinnamomum osmophloeum*	AtTPS2	NP_193406
CtGES	Q8GUE4	*Cinnamomum tenuipile*	AtTPS3	NP_567511
LcTPS2	G0Y7D2	*Litsea cubeba*	AtTPS4	NP_564772
LiS-LINS	AXF50410	*Lavandula x intermedia*	AtTPS5	NP_179904
LpPIN	T1RR71	*Lavandula pedunculata*	AtTPS6	NP_177165
FaPIN	O23945	*Fragaria vesca*	AtTPS7	NP_193753
MdPIN	NP_001281061	*Malus domestica*	AtTPS8	NP_193754
CsLINS	AHC54051	*Coriandrum sativum*	AtTPS9	NP_193756
CsTPS35	QLC36840	*Cannabis sativa*	AtTPS10	NP_179998
LcTPS1	G0Y7D1	*Litsea cubeba*	AtTPS11	NP_199276
LnTPS1	AKQ19357	*Laurus nobilis*	AtTPS12	NP_193064
Cslis	QNI69163	*Camellia sinensis*	AtTPS14	NP_176361
Amlis	ABR24418	*Antirrhinum majus*	AtTPS15	NP_189564
ZpTPS3	BBD88590	*Zanthoxylum piperitum*	AtTPS16	NP_189555
PdTPS1	XP_034211271	*Prunus dulcis*	AtTPS17	NP_188067
CiCaMS	QNV69587	*Cinnamomum camphora*	AtTPS18	NP_188070
SBS	O81192	*Salvia officinalis*	AtTPS19	NP_188072
LaBPPS	AJW68082	*Lavandula angustifolia*	AtTPS21	NP_197784
PdBPPS	ATY48638	*Phyla dulcis*	AtTPS22	NP_174635
WvBPPS	AWW87313	*Wurfbainia villosa*	AtTPS23	NP_189210
PLPIN	KU187411	*Paeonia lactiflora*	AtTPS24	NP_189209
PpCPS/KS	XP_024380398	*Physcomitrium patens*	AtTPS25	NP_189587
AtEKS	NP_178064	*Arabidopsis thaliana*	AtTPS26	NP_176776

SrKS1	Q9XEH9	*Stevia rebaudiana*	AtTPS27	NP_189210
CmKS	XP_022968895	*Cucurbita maxima*	AtTPS28	NP_175312
AtKS	AAC39443	*Arabidopsis thaliana*	AtTPS29	NP_001319127
CcTPS2	QNV69588	*Cinnamomum camphora*	AtTPS30	NP_189746
LnTPS2	AKQ19358	*Laurus nobilis*		
ZpTPS1	BBD88588	*Zanthoxylum piperitum*		
SgTPS	UOW66203	*Sindora glabra*		
SmSTPS	F6M8H7	*Santalum murrayanum*		
SvTPS15	XP_049345898	*Solanum verrucosum*		
OvTPS3	E2E2N8	*Origanum vulgare*		
SsTPS12	XP_049379744	*Solanum stenotomum*		
SLTPS9	NP_001234055	*Solanum lycopersicum*		
ZaTPS	UJH94381	*Zanthoxylum ailanthoides*		
SLTPS31	NP_001239040	*Solanum lycopersicum*		
CbTPS1	QTW43990	*Cinnamomum burmanni*		
PmTPS2	AWK77755	*Persicaria minor*		
PdTPS2	QEE82240	*Prunus dulcis*		
AkTPS2	BBO53955	*Angelica keiskei*		
TwTPS	AWV55521	*Tripterygium wilfordii*		
GbTPS3	KAB2044572	*Gossypium barbadense*		

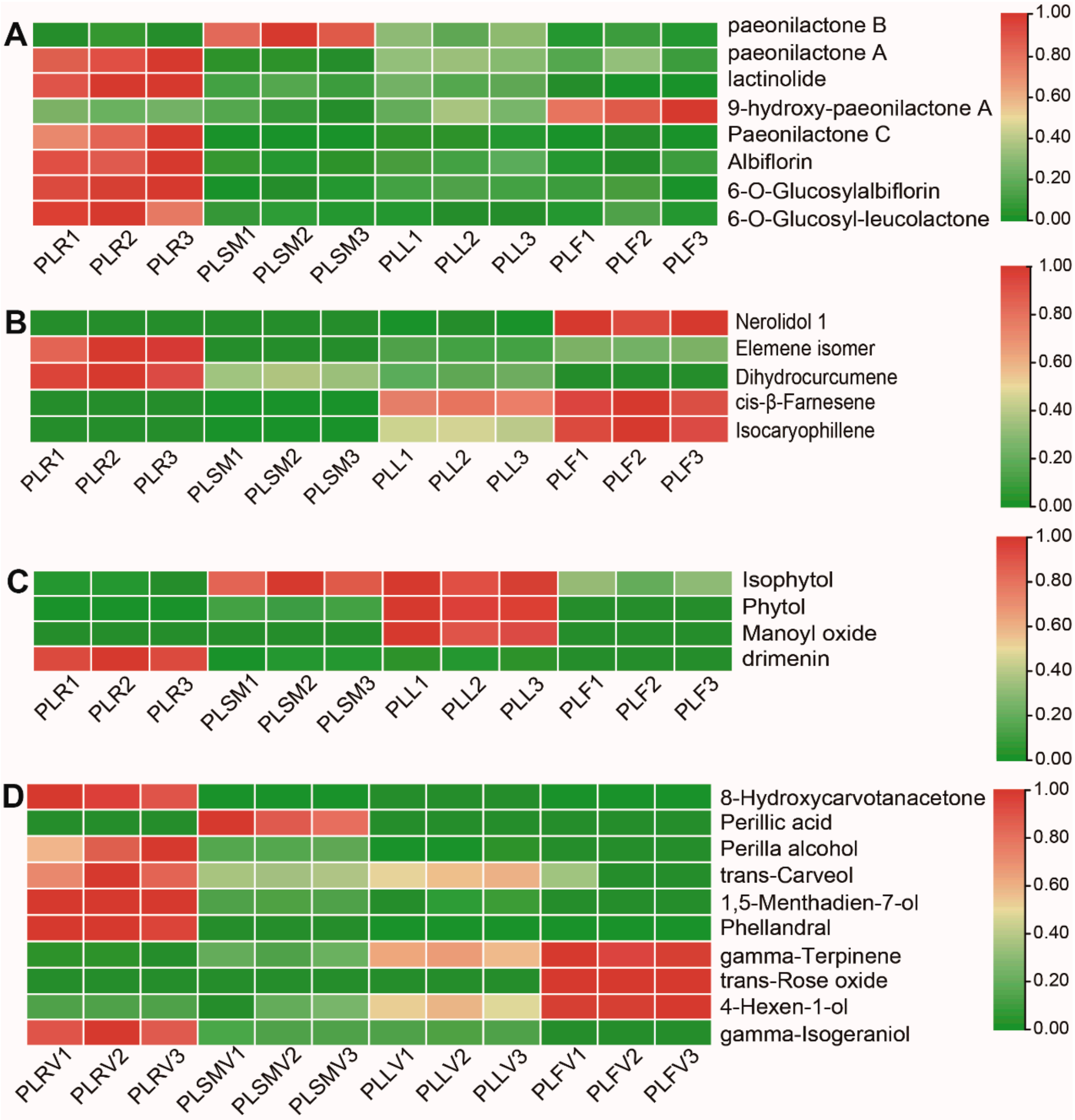

Figure S1 Metabolome results for different tissues of *P. lactiflora*. A: Heatmap analysis of *P. lactiflora* about different tissue parts of Non paeoniflorin derivatives. B: Heatmap analysis of *P. lactiflora* about different tissue parts of sesquiterpenoids. C: Heatmap analysis of *P. lactiflora* about different tissue parts of diterpenoids. C: Heatmap analysis of *P. lactiflora* about different tissue parts of Non Pinane Monoterpenoids.

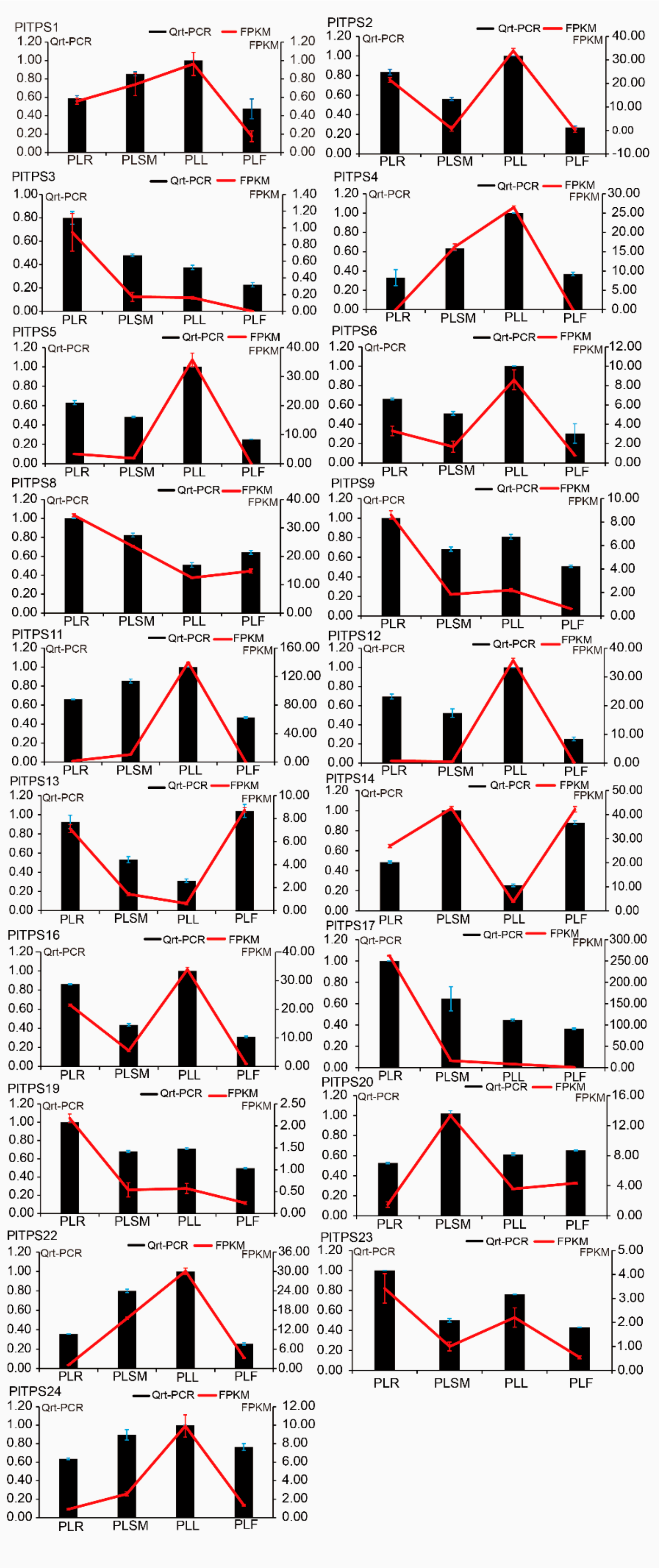

Figure S2 Relative expression level of *P. lactiflora* of PlTPSs by RT-qPCR and the reads per kilobase million value from the *P. lactiflora* transcriptome.

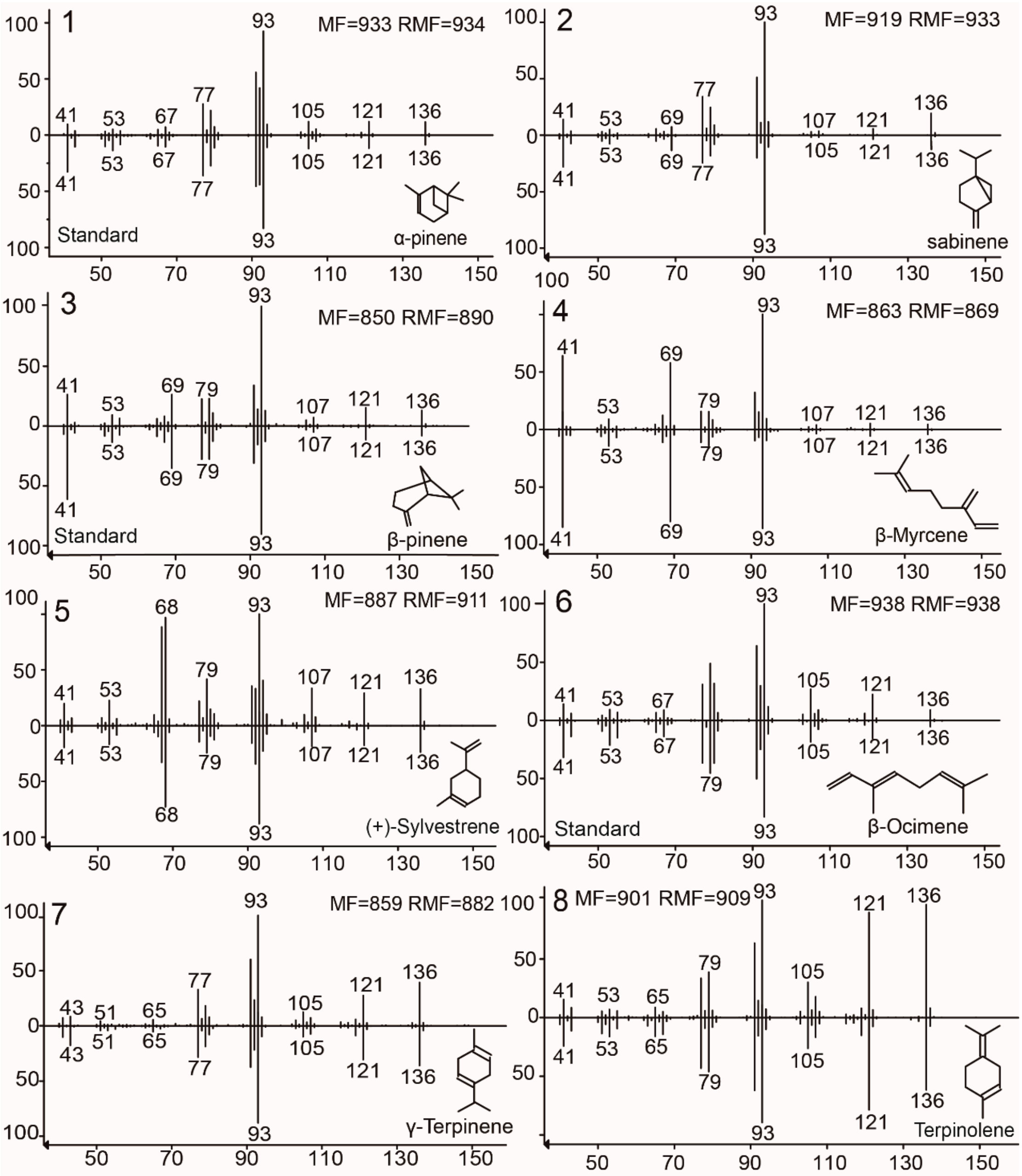

Figure S3 GC–MS analysis of *P. lactiflora* Class I terpene synthase in vitro assays with engineered yeast that can produce GPP. The m/z of products were matched with NIST mass spectral library for identification of a potential terpenes.

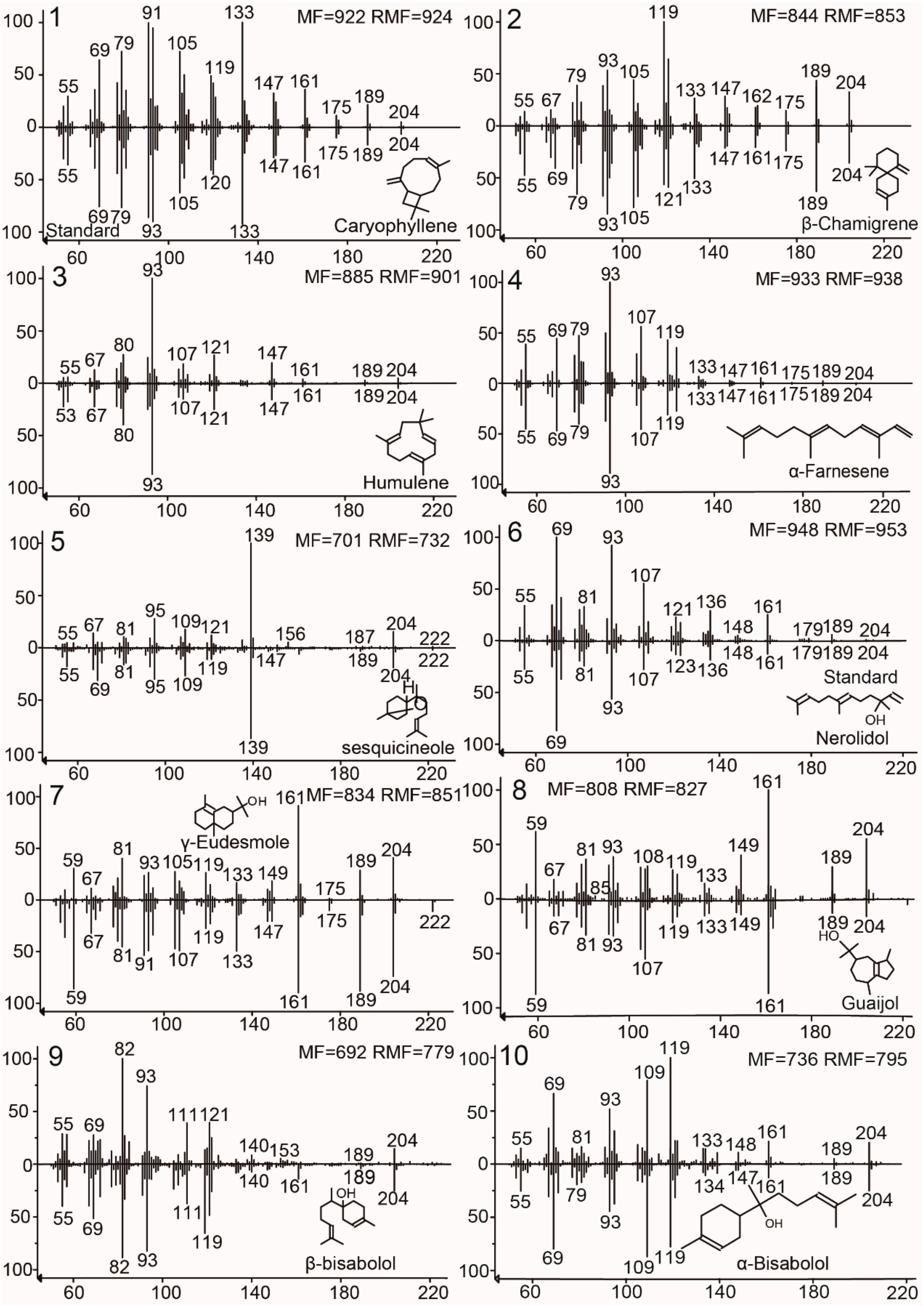

Figure S4 GC–MS analysis of *P. lactiflora* Class I terpene synthase in vitro assays with engineered yeast that can produce FPP. The m/z of products were matched with NIST mass spectral library for identification of a potential terpenes.
